# Quantitative assessment of Pb sources in isotopic mixtures using a Bayesian mixing model

**DOI:** 10.1038/s41598-018-24474-0

**Published:** 2018-04-18

**Authors:** Jack Longman, Daniel Veres, Vasile Ersek, Donald L. Phillips, Catherine Chauvel, Calin G. Tamas

**Affiliations:** 1 Department of Geography and Environmental Sciences, Northumbria University, Newcastle-upon-Tyne, NE1 8ST United Kingdom; 2 School of Ocean and Earth Sciences, University of Southampton, National Oceanography Centre, Waterfront Campus, Southampton, SO14 3ZH United Kingdom; 3 Romanian Academy, Institute of Speleology, Clinicilor 5, Cluj-Napoca, Romania; 4 EcoIsoMix.com, Corvallis, Oregon, USA; 5 CNRS, Université Grenoble Alpes, Institut des Sciences de la Terre, UMR 5275 CNRS Grenoble, France; 6 Institut de Physique du Globe de Paris, Université Sorbonne Paris Cité, CNRS UMR 7154 Paris, France; 7 Faculty of Biology and Geology, University Babeş-Bolyai, 1M. Kogălniceanu str., 400084 Cluj-Napoca, Romania

## Abstract

Lead (Pb) isotopes provide valuable insights into the origin of Pb within a sample, typically allowing for reliable fingerprinting of their source. This is useful for a variety of applications, from tracing sources of pollution-related Pb, to the origins of Pb in archaeological artefacts. However, current approaches investigate source proportions via graphical means, or simple mixing models. As such, an approach, which quantitatively assesses source proportions and fingerprints the signature of analysed Pb, especially for larger numbers of sources, would be valuable. Here we use an advanced Bayesian isotope mixing model for three such applications: tracing dust sources in pre-anthropogenic environmental samples, tracking changing ore exploitation during the Roman period, and identifying the source of Pb in a Roman-age mining artefact. These examples indicate this approach can understand changing Pb sources deposited during both pre-anthropogenic times, when natural cycling of Pb dominated, and the Roman period, one marked by significant anthropogenic pollution. Our archaeometric investigation indicates clear input of Pb from Romanian ores previously speculated, but not proven, to have been the Pb source. Our approach can be applied to a range of disciplines, providing a new method for robustly tracing sources of Pb observed within a variety of environments.

## Introduction

Lead (Pb), a toxic, non-essential metal for life, has been an important commodity in our technological development^1^ and one of the most persistent anthropogenic pollutants through time^2,3^. Lead may be released directly from the mining and smelting of Pb-containing ores, and more recently, through the extensive use of Pb-containing fuels, batteries, paints and other commodities^4^. Lead is among the best-studied metal pollutants, due in part to its toxicity, even at low levels, to multiple organ systems^5^, and its particular danger to young children and developing foetuses^6^. As such, understanding the past interplay between natural and anthropogenic sources, sinks and cycles of Pb is vital, as evidence suggests humans have greatly impacted its natural biogeochemical cycle^7,8^. Records of pollution indicate anthropogenic influences as far back as the Bronze Age^3,9,10^. Particularly large amounts of Pb were released into the atmosphere during the Roman period in Europe, with the geochemical imprint of Pb pollution especially quantifiable in peat records^7,9,11,12^. Evidence of such activity may even be observed in Greenland ice, clear indication of the persistence of Pb pollution even in such pristine environments^13,14^. Further anthropogenic atmospheric Pb enrichment has been observed in records located close to centres of mining in Medieval times^9,15,16^, and upon the introduction of Pb-containing gasoline in the early 20^th^ century, virtually across the globe^10,17–20^, even as far from mining centres as Antarctica^21^.

Knowledge of the pollution activity at the sites of mining and smelting, and assessment of contamination levels at different sites is not always sufficient for firm conclusions regarding Pb provenance and contributions from changing sources or contributors through time. In an effort to clarify such questions, Pb isotopes have been long used as ‘fingerprints’ of the source of Pb recorded in environmental archives^3,4,12^. Within the natural environment, Pb is mainly present as four stable isotopes: ^208^Pb (≈52%), ^206^Pb (≈24%), ^207^Pb (≈23%) and ^204^Pb (≈1%)^4^. ^204^Pb represents the only Pb isotope not a product of radioactive decay chains, with ^206^Pb, ^207^Pb and ^208^Pb originating in the decay chains of ^238^U, ^235^U and ^232^Th, respectively^4,22^. Thus, the ratios of different Pb isotopes depend on the concentrations of U, Th and Pb in various source materials, the length of the half-lives of the parent isotopes^23^ and the amount of time that passed. In environmental science, Pb isotopic data are commonly presented as ratios, with ^206^Pb/^207^Pb, ^208^Pb/^206^Pb typically used^24^. Analyses of ore bodies have indicated the variability of Pb isotope ratios depends on the type of ore, age, and style of mineralization^25^. A simple example is the difference observable between the ^206^Pb/^207^Pb ratio of very old ores such as the Broken Hill ore (formed 1.6–1.8 billion years ago, and recently used in European leaded petrol), with ^206^Pb/^207^Pb = 1.05–1.1^26–28^ whilst younger ores (most Jurassic to Miocene ores) fall within the range ^206^Pb/^207^Pb = 1.18–1.25^29^. This is because when ores form, their Pb isotope ratios reflect their crustal source, a reservoir whose Pb isotopic ratios increase slowly with time due to the radioactive decay of U and Th. After ore formation, no radiogenic growth occurs because the ores contain little or no U or Th. Thus, the Pb isotopic ratios remain identical to the crustal composition at time of ore formation^30,31^.

Crucially for archaeometallurgical inferences, the Pb isotopic ratios are not severely altered by physical or chemical fractionation processes during ore processing, smelting, and casting, although some minor variations may occur^32^. Therefore it can be assumed the Pb isotope values of the pollution-related Pb are very similar to those of the parent ore bodies^33^. As such, precise isotopic ratios may be used to ‘link’ Pb deposited in a substrate (e.g. bogs, lakes, ice) to a certain ore^4,33^. It has been demonstrated that records of Pb concentration from sedimentary archives such as peat bogs, lake sediments and ice cores may retain the isotopic signature of the original source of Pb, since once deposited within such environments, Pb is largely immobile^34^. Such work has clearly indicated the impact of metal pollution from both local sources^35^, and long range atmospheric transport^9,10,14^ through time. However, the signal recorded within a bog or lake represents a mixture of many local, regional and extraregional inputs, including contribution from natural sources (dust, biomass burning, volcanoes, etc) and disentangling these has proven challenging^36^.

**Figure 1:**
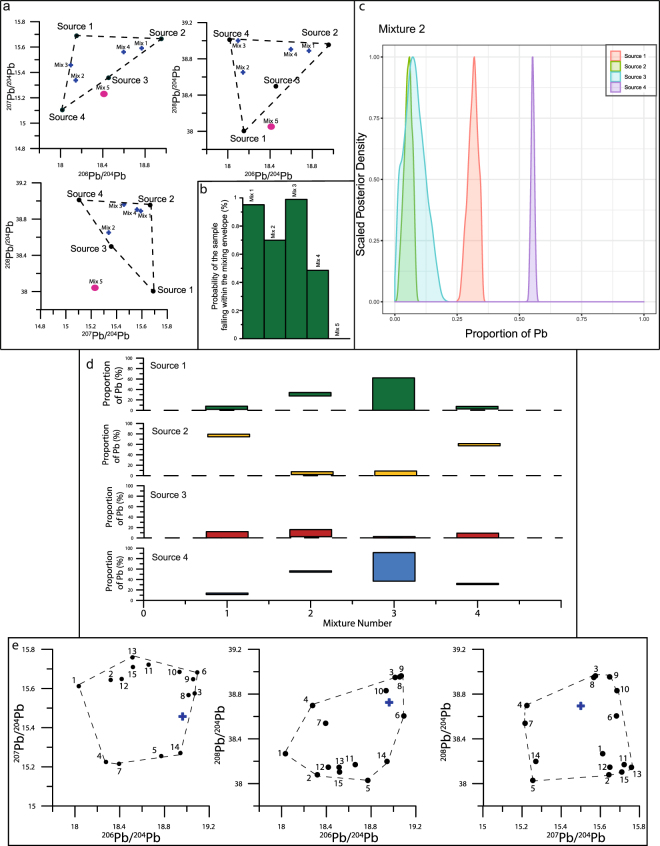
Theoretical examples. Figure displays input (**a**) in the form of convex hulls; three panels, each displaying a three-isotope plot with the corresponding sources and mixtures to be modelled. Dashed black lines have been added to outline the mixing envelope, as defined by the mean values for each source (black circles). Also displayed are mixtures which fall within the envelope blue crosses) and one which does not (pink circles). Panel (b) displays the output from mixing envelope modelling, and the probability of each sample falling within the mixing envelope (%). (**c**) Is an example posterior density graph as output by MixSIAR, here from the model of mixture 2. Each field corresponds to the probability of a given source to provide the Pb present in the mixture. (**d**) Is the overall output of all four mixtures modelled in this example. In each case, the rectangle indicates the range of outputs, with the upper and lower bounds signifying the 2.5% and 97.5% credible intervals. (**e**) Displays convex hulls of theoretical example 2. Black circles denote the sources used to model the mixture (blue cross).

Moreover, without a modelling component in interpreting Pb records from sedimentary archives, typical approaches may only be used to indicate likely sources, rather than infer quantitative apportionments. Traditionally, interpretation of Pb isotopic ratios is performed via a 3-isotope, or 4-isotope plots (see Fig. 1a), and comparing the location of the sink mixture data (the isotope signal recorded within the archive) to the isotopic signatures of known sources. Attempts have been made to model this sink mixture as a way of quantitatively assessing the relationships between the Pb recorded within a sediment and its source^37,38^. Until now however, Pb isotope mixing models have generally been limited to considering either two^37,39,40^ or three^38^ potential sources. This approach is adequate in a very well determined system, where only a few possible sources may be considered, or only a couple of broad sources are of interest^37,39^. This approach allows identification of very general source inputs, such as anthropogenic vs lithogenic Pb. Other studies have been able to distinguish petrol-derived Pb^41^, or local ore-derived Pb from other anthropogenic sources^42^, but the lack of further source consideration precludes defined provenance of such Pb to an ore body or mining region in a complex mixture^37^. This is due to the limitations related to the models and the Pb isotope systems themselves, where generally only two or three isotope ratios (n) are interpreted, and thus limiting such models to n + 1 sources. These types of models, therefore, may be useful in a general overview, but the complexity of potential Pb inputs, particularly in an anthropogenically polluted world, cause such approaches to be likely oversimplifications, with only limited conclusions possible^37^.

In the field of biology, mixing models have long been used to apportion large numbers of prey sources within, for example, a predator’s diet^43–45^, from stable isotope compositions using mixing models such as IsoSource^46^ and MixSIAR^47,48^. When IsoSource was developed, an initial attempt to apply it to pollution sourcing via Pb isotopes was made^46^. However, to our knowledge, little further work to further apply this method to the field has been done^49^, likely due to diffuse and poorly constrained outcome sources. Other approaches to Pb isotope modelling have been applied, with Euclidean distance calculation used to determine potential source(s) for archaeological artefacts^50,51^. This method, however, simply indicates the most likely ore fields (or specific mines) within a specific database.

Application of more complex models to Pb isotope tracing, therefore, could provide a novel approach to source apportionment, with potentially a better understanding of past pollution input than might be achieved solely through 3-isotope graphing or Euclidean distance modelling. In practice, this may be able to produce more reliable tracing of Pb in the environment, because far more potential sources can be considered, and less simplification of the system is necessary. This approach could be applied to the interpretation of indirect records (such as from peat, lake sediments, ice) of historical pollution, modern pollution, and even the origin of metal used in archaeological artefacts.

Here we propose a new approach, using a number of real-world examples. First we outline the principles, using a pair of theoretical models. We then apply the method to pre-anthropogenic samples from a peat bog in North-western Spain^52^ to track the changing influence of Saharan dust on the Pb isotope ratio. A second real-world example utilises data from the same site but from a period of heavy pollution (namely the Roman period) to trace likely ore sources of Pb deposited within the bog. Finally, we exploit the ability of the model to consider a very large number of sources, and investigate the origin of Pb in a litharge roll discovered within ancient mining works in Rosia Montana mine, Romania, and dating back to the Roman period^53^.

## Materials and Methods

### Mixing Models

As the focus of the study is on the application of complex mixing models to Pb isotopes from bulk sediment samples and artefacts, we do not outline the theory behind simple binary, or ternary mixing approaches^30,37,38^. For disentangling multiple sources we considered two possible approaches: 1) IsoSource, a mass balance-based multi-source mixing model^46^ and 2) MixSIAR^47^, the latest iteration of a series of Bayesian mixing models^48,54^.

IsoSource examines all possible source contribution configurations, at user-defined intervals (e.g. 1%). These calculations rely upon the following equations, expressed below for a configuration with one isotope system and three sources:

1 $${X}_{M}={f}_{A}{X}_{A}+{f}_{B}{X}_{B}+{f}_{C}{X}_{C}$$

2 $$1={f}_{A}+{f}_{B}+{f}_{C}$$

where:

*X* = Isotopic signature of the mixture (M) or sources (A,B,C).

*f*_*A*_, *f*_*B*_, *f*_*C*_ = Proportion of Pb from each source.

*X*_*A*_, *X*_*B*_, *X*_*C*_ = Source isotopic signatures.

This is an underdetermined system containing three unknowns and two equations, with no unique solution^46^. However, the predicted mixture signature for each source combination can be compared to the observed mixture signature; if it matches, then mass balance is achieved and that source combination is a feasible solution. Because combinations can only be examined in discrete steps, a small tolerance for deviation from exact matches is allowed^46^. These equations make interpretation of datasets with n isotope systems and more than n + 1 sources possible^45,46,49^. Outputs are generally presented as range values, to ensure that all potential combinations are reported^45^.

IsoSource may provide valuable model output, and prior to development of Bayesian isotopic mixing models, it was the main approach for food web studies^55^. However, IsoSource uses only mean isotopic signatures for sources and mixtures and does not directly account for variability in source and mixture ratios, including sampling and measurement errors. Because ore bodies are typically heterogeneous, ascribing a single value is a simplification. Complex models have recently been developed which do incorporate these uncertainties within the framework of rigorous Bayesian statistics, utilising the same basic principles as outlined in equations 1 and 2^48^. Put simply, this approach allows the user to incorporate prior information about the system, including analytical error and source heterogeneity, and using a natural distribution (i.e., Dirichlet distribution^56^) allows the model to develop solutions, in terms of percentage contributions which sum to unity. The Dirichlet-defined distribution is intentionally vague, allowing for the model to be driven primarily by the data input by the user. Model fits are developed via many Markov Chain Monte Carlo (MCMC) simulations, which produce simulations of plausible source proportions based upon the data, and probability densities. Any mixtures not probabilistically consistent with the data are discarded, and mixtures developed at the end of the run are required to be close to the early ones in terms of proportion, causing a Markov chain to be converged^48^. These, when combined with the prior information (e.g. results of previous studies if available) produce posterior distributions which are true probability distributions for each source^48^.

Models built around a Bayesian approach include MixSIAR^47^, which is a combination of MixSIR^54^ and SIAR^48^, as well as FRUITS^57^, IsotopeR^58^, and others, which are slightly varied approaches to the same problem. Here we propose an approach for applying a state-of-the-art Bayesian stable isotope mixing model (MixSIAR) to pollution sourcing and artefact tracing via Pb isotopes. “MixSIAR” is a package which runs in the R framework. Details on downloading and setting up the package may be found at https://cran.r-project.org/web/packages/MixSIAR/index.html.

### Outline of the approach, with two theoretical applications

#### Pre-Modelling Preparation

Prior to attempting data modelling, the user must ensure that all potential sources (mining regions active or probably active during the period of study, active smelters, potential natural Pb sources) that may have contributed to the Pb isotope signal at the study site are considered in the analysis. It should be noted that for pollution or archaeological applications, the approach is likely complicated by the fact that potential sources will have changed over time, or that ores once actively mined may have been exhausted. When developing source lists, all potential sources (with sufficient data available) must be considered, regardless of locality, due to the reality of long-range transport of Pb^14,59^. Further, for pollution tracing of sediment samples, a local ‘natural’ Pb isotope signal should be introduced to the model, to ensure any such local input (e.g. erosion products) of Pb is considered.

All data must be collated, and a database of all signatures prepared. As previously suggested, Pb isotope ratios relative to ^204^Pb should be used (i.e. ^208^Pb/^204^Pb, ^207^Pb/^204^Pb and ^206^Pb/^204^Pb). This allows for as much of the possible variability in the isotopic signature to be considered during the modelling, and because each isotope originates from a different decay chain^23^, a degree of independence between the three ratios. If several analyses are available for the same site, these data should be averaged before standard deviations are calculated. This allows for errors related to either measurement uncertainty or source heterogeneity to be incorporated into the model.

Because MixSIAR uses standard deviations and means to develop models, multivariate normal distributions of the isotopic compositions of ores are assumed. Previous work appears to suggest this is not necessarily the case^60,61^. This is typically ascribed to the complex origins of most ore bodies, in some cases with multiple depositional phases of Pb or polymetallic (Pb containing) ores at the same site common, resulting in multimodal distributions and a degree of kurtosis. However, it has been argued such multimodal distributions may, when enough data are presented, still be representative of multivariate normal (MVN), as multiple depositional events may even one another out, averaging the data^62^. Discussion of this point may be disputable as all such studies investigated the behaviour of the 3-dimensional mixing space developed between the ratios ^206^Pb/^204^Pb, ^207^Pb/^206^Pb and ^208^Pb/^206^Pb, and we encourage modelling of the envelope of isotopes normalised to ^204^Pb (^208^Pb/^204^Pb, ^207^Pb/^204^Pb and ^206^Pb/^204^Pb).

To determine the distribution of the ore bodies considered here, and to ensure MiXSIAR is an appropriate approach, we have performed a number of tests investigating the nature of their isotopic distributions. First, using univariate Shapiro-Wilk and Kolmogorov-Smirnov tests, we determine the normality of each isotope ratio in each field individually, prior to investigation of MVN of the three-ratio system via further tests (Mardia, Henze-Zirkler and Royston). All univariate analysis was performed on SPSS 22, and multivariate analysis using the package “MVN” in R^63^.

Results (see Table 1) indicate normality is typical for the distributions of ^206^Pb/^204^Pb (78% of ore bodies), ^207^Pb/^204^Pb (82%) and ^208^Pb/^204^Pb (69%). MVN examinations (Table 2) indicate most ore bodies may be considered MVN via at least one of the tests (78%), with smaller databases (e.g. Apuseni Porphyry, Mazarron and Oberlausitz) more likely to be MVN, but with larger databases also displaying MVN (e.g. Almeria, Gaul and Valsugana VMS). Notably, some complex and heterogeneous ore bodies (e.g. Apuseni Epithermal), are not MVN, indicating multiple mineralization phases, as observed at Rosia Montana, part of the Apuseni Epithermal ore body^53^, do not even out the isotope distribution range. The MVN distribution of a majority of ore bodies, and the uncertain nature of a number more appear to indicate an approach, which assumes MVN may be applicable. Crucially, the outcomes from these tests are inconclusive, neither proving nor disproving categorically an MVN nature of lead isotopes. As a result, an approach, which applies means and SDs appears the simplest and most appropriate target for such analyses.

**Table 1:** Univariate normality tests for all ore bodies used to construct models in this work.

Univariate Normality Tests													
		Shapiro-Wilk						Kolmogorov-Smirnov					
		206Pb/204Pb		207Pb/204Pb		208Pb/204Pb		206Pb/204Pb		207Pb/204Pb		208Pb/204Pb	
Field	n	W	p-value	W	p-value	W	p-value	W	p-value	W	p-value	W	p-value
Almeria	23	0.9198	***0***.***0752***	0.909	***0***.***451***	0.8924	0.021	0.1557	***0***.***179***	0.1906	0.0365	0.193	0.0323
Andalusia	11	0.6362	0.0001	0.9203	***0***.***3591***	0.9803	***0***.***9666***	0.4103	0	0.1584	***0***.***6786***	0.1523	***0***.***7347***
Apuseni Epithermal	40	0.9594	***0***.***1709***	0.7428	0	0.755	0	0.1291	***0***.***0999***	0.2542	0	0.2528	0
Apuseni Porphyry	7	0.9539	***0***.***772***	0.9176	***0***.***488***	0.9508	***0***.***7466***	0.1667	***0***.***8668***	0.2432	***0***.***323***	0.1918	***0***.***6991***
Bohemia	11	0.5587	0	0.7152	0.0013	0.6864	0.0006	0.4654	0	0.3388	0.0018	0.3933	0.0001
Central Erzgebirge	12	0.957	***0***.***7344***	0.8987	***0***.***1783***	0.915	***0***.***2794***	0.1321	***0***.***8541***	0.1991	***0***.***2581***	0.17777	***0***.***4297***
Dobrogea*	5	0.8024	***0***.***1065***	0.7881	***0***.***0826***	0.8	***0***.***1022***	n/a	n/a	n/a	n/a	n/a	n/a
Eastern Erzgebirge	14	0.6506	0.0002	0.7722	0.0032	0.588	0.0001	0.3206	0.0007	0.2686	0.011	0.3103	0.0013
Gaul	32	0.9562	***0***.***2315***	0.988	***0***.***9745***	0.8369	0.0003	0.1081	***0***.***4708***	0.107	***0***.***4866***	0.258	0
Huelva	10	0.9581	***0***.***7784***	0.9722	***0***.***9132***	0.9583	***0***.***7806***	0.196	***0***.***4082***	0.1643	***0***.***6896***	0.1413	***0***.***8702***
Ibias	6	0.9014	***0***.***4176***	0.908	***0***.***4555***	0.9057	***0***.***4424***	0.2337	***0***.***4923***	0.3	***0***.***146***	0.2061	***0***.***6911***
Leonese Zone	8	0.9263	***0***.***5198***	0.5252	0	0.9058	***0***.***3678***	0.2084	***0***.***4736***	0.4746	0	0.2499	***0***.***2062***
Mazarron	7	0.9039	***0***.***3973***	0.9698	***0***.***891***	0.9462	***0***.***7097***	0.2089	***0***.***5668***	0.186	***0***.***7423***	0.2333	***0***.***3869***
Northern Erzgebirge	6	0.886	***0***.***3374***	0.8532	***0***.***2049***	0.9134	***0***.***4884***	0.3296	***0***.***0802***	0.2802	***0***.***2204***	0.3	***0***.***416***
Oberlausitz*	5	0.8311	***0***.***1707***	0.8965	***0***.***4141***	0.9713	***0***.***8497***	n/a	n/a	n/a	n/a	n/a	n/a
Panagyurishte	17	0.7621	0.0009	0.9299	***0***.***2431***	0.9339	***0***.***2811***	0.3007	0.0004	0.1303	***0***.***6623***	0.1789	***0***.***186***
Reocin	11	0.9551	***0***.***7294***	0.9485	***0***.***6504***	0.9622	***0***.***8103***	0.1463	***0***.***7877***	0.148	***0***.***7733***	0.1414	***0***.***8259***
Shropshire	12	0.9288	***0***.***3984***	0.8671	***0***.***0711***	0.8109	0.0131	0.1712	***0***.***4898***	0.2363	***0***.***0868***	0.2863	0.0121
Slovakia*	5	0.925	***0***.***5653***	0.9779	***0***.***8898***	0.8647	***0***.***2772***	n/a	n/a	n/a	n/a	n/a	n/a
Suior, Baia Sprie, Cavnic, Herja	20	0.915	***0***.***081***	0.973	***0***.***811***	0.953	***0***.***423***	0.176	***0***.***105***	0.091	***0***.***2***	0.194	0.047
Valsugana VMS	24	0.9125	***0***.***0561***	0.9383	***0***.***1655***	0.9621	***0***.***5078***	0.1769	***0***.***0602***	0.2045	0.0135	0.1317	***0***.***3784***
Vogtland	11	0.9128	***0***.***3004***	0.8804	***0***.***1319***	0.9371	***0***.***5213***	0.1654	***0***.***6121***	0.2364	***0***.***1167***	0.218	***0***.***1951***
Western Balkans	15	0.7901	0.0038	0.9685	***0***.***8563***	0.9539	***0***.***6224***	0.2939	0.0018	0.1453	***0***.***586***	0.1542	***0***.***4887***

For both Shapiro-Wilk and Kolmogorov-Smirnov tests, W indicates the associated test statistic, whilst the p-value indicates the significance of this statistic. Those highlighted in bold and italicised are significant at 5%, indicative of a normal distribution. Raw data may be found in Tables SI 1–3.

^*^Insufficient data points for Kolmogorov-Smirnov test.

**Table 2:** Multivariate normality (MVN) tests results for each ore body mentioned in this work.

	Multivariate Normality Tests									
		Mardia					Henze-Zirkler		Royston	
Field	n	g1p	p-value (skewness)	g2p	p-value (kurtosis)	p-value (small sample skewness)	HZ	p-value	H	p-value
Almeria	23	7.0921	0.0037	14.1732	0.7233	0.0004	1.6457	0	7.4357	***0***.***0591***
Andalusia	11	4.7755	***0***.***6328***	11.3022	0.2858	***0***.***2864***	0.719	***0***.***0643***	7.8207	0.0172
Apuseni Epithermal	40	6.3177	0	19.3634	0.0129	0	3.5644	0	29.558	0
Apuseni Porphyry	7	5.5074	***0***.***8548***	10.9011	0.3594	***0***.***3966***	0.482	***0***.***3388***	0.278	***0***.***9197***
Bohemia	11	8.1672	***0***.***1914***	15.1959	0.9549	0.0249	1.0391	0.0016	16.6741	0.0001
Central Erzgebirge	12	5.2404	***0***.***4756***	13.2149	0.5889	***0***.***1741***	0.7034	***0***.***0857***	2.2641	***0***.***3388***
Dobrogea	5	6	***0***.***9473***	9	0.2733	***0***.***4405***	0.4185	***0***.***3934***	0.966	***0***.***1475***
Eastern Erzgebirge	14	13.8406	0.0009	20.5215	0.0691	0	1.2063	0.0003	25.26228	0
Gaul	32	2.7223	***0***.***17***	13.2911	0.3851	***0***.***0945***	1.3579	0.0002	14.959	0.0019
Huelva	10	7.8091	***0***.***3046***	14.5425	0.9003	0.0589	0.7343	***0***.***0572***	0.0555	***0***.***9845***
Ibias	6	6.7523	***0***.***8456***	10.5542	0.3641	***0***.***2848***	0.4541	***0***.***3599***	0.5281	***0***.***6308***
Leonese Zone	8	11.74429	***0***.***187***	15.2725	0.9475	0.0074	1.1164	0.0003	14.3898	0.0024
Mazarron	7	5.2806	***0***.***8717***	10.5677	0.3216	***0***.***4334***	0.4279	***0***.***5085***	0.4688	***0***.***8655***
Northern Erzgebirge	6	6.6793	***0***.***8503***	10.4113	0.3489	***0***.***2935***	0.4902	***0***.***2626***	1.4126	***0***.***4675***
Oberlausitz	5	6	***0***.***9473***	9	0.2733	***0***.***4405***	0.4185	***0***.***3935***	1.7835	***0***.***6483***
Panagyurishte	17	4.2847	***0***.***3253***	15.01265	0.9963	***0***.***1365***	1.1477	0.001	13.9998	***0***.***0592***
Reocin	11	2.3708	***0***.***9495***	11.076	0.2573	***0***.***8196***	0.4702	***0***.***5462***	0.168	***0***.***9151***
Shropshire	12	4.9576	***0***.***5237***	13.0204	0.5489	***0***.***2116***	0.815	0.0262	8.3212	0.0288
Slovakia	5	6	***0***.***9473***	9	0.2733	***0***.***4405***	0.4185	***0***.***3934***	0.4844	***0***.***7198***
Suior, Baia Sprie, Cavnic, Herja	20	9.6058	0.0007	20.6344	0.0249	0	1.3159	0.0001	3.8071	***0***.***1813***
Valsugana VMS	24	2.5574	***0***.***4579***	12.5389	0.2813	***0***.***2972***	1.4892	0	3.4752	***0***.***1304***
Vogtland	11	3.3178	***0***.***8531***	10.1964	0.1655	***0***.***5973***	0.7298	***0***.***0575***	1.4207	***0***.***2795***
Western Balkans	15	8.9698	0.0216	18.6692	0.2101	0.0017	1.0834	0.0017	7.8609	***0***.***0512***

Three tests (Mardia, Henze-Zirkler and Royston) are presented. For Mardia’s multivariate normal test, g1p is the statistic of skewness and g2p the statistic of kurtosis, and p-values indicate the significance of these values. Also presented is the p-value of small sample skewness, a further significance value, developed using empirical critical values for small sample sizes (n < 50^87^). For both Henze-Zirkler and Royston tests only a single statistic is presented, HZ and H, respectively. All p-values significant at 5% are in bold and italicised, indicating multivariate normal distribution for that ore body.

Consequently, grouping, one of the most important parts of this analysis, should be approached robustly and can be done *a priori*, or *a posteriori*^64^. *A priori* grouping should be carried out to align sources (prior to the analysis), which are very similar in isotopic signature, or are all from the same ore field, but ensuring any MVN is not lost. Significance tests^65^ may be performed, to ensure that the groups are significantly different. If not, they should be grouped together.

### Pre-Run Checks

Prior to running the model, checks must be performed to ensure the data for the unknown mixture fall within the mixing envelope^46^ as determined via the sources identified. Due to the nature of MixSIAR^47^ results are produced even when these are not realistic. The mixing envelope can be visualized in each 2-dimensional cross-section of the isotope space (see Fig. 1a). To be explained as simple mixtures of these sources, mixtures must fall within the space defined by the various sources. If they do not, then it must be considered that there is an issue in the working hypotheses: either errors during measurement, or a significant source of Pb has been overlooked in the analysis^46^. Because Bayesian modelling incorporates uncertainties (in this case means and standard deviations), a further Bayesian modelling tool may be used to ascertain exactly which samples fall within the mixing polygon^66^. From this further model, any samples which fall within the polygon in <5% of iterations should be discarded. Further, if a significant number are not constrained within the polygon, this is an indication the model being applied is not correct and should be reconsidered. An instance of this issue may be observed from the example in Fig. 1a, where one sample (shown by a red cross) falls outside the designated potential sources. Model results confirm this (Fig. 1b), indicating there is 0% chance of the sample falling within the polygon, and therefore it is not interpreted.

### Post-Run Checks

Once all the preparation steps have been completed, the model employs during its run Markov Chain Monte Carlo (MCMC) statistics, which investigate probability distributions for variables (in this example the proportion of Pb originating from each source). From these ‘posterior’ distributions (see Fig. 1c), descriptive statistics including mean, standard deviation and range may be produced. It must be noted such outputs are only indicative of proportion of Pb from each source.

To ensure meaningful results, the MCMC chains must be long enough for convergence to be achieved. Convergence of the data may be checked via two diagnostics post-run: Gelman-Rubin and Gewecke tests^47,65^. The Gelman-Rubin test requires at least 1 chain, and values will be near 1 at convergence, therefore high values indicate non-convergence. The Gewecke test compares the two halves of the MCMC chain and develops z-scores to determine convergence. High z-scores are an immediate indicator of non-convergence, and so a longer run must be performed^47^.

In practice, with many sources and three isotope ratios, MixSIAR generally must be set to either ‘long’ or ‘very long’ MCMC chains to achieve satisfactory convergence. However, this may vary, and so it is a valuable exercise checking the post-run diagnostics, and in running the model at various lengths. For all models discussed here, ‘long’ or ‘very long’ MCMC chains have been run.

### A Posteriori Grouping

When only a small number of well-constrained sources are considered, valuable results may be derived from the raw model outputs (Fig. 1d). However, Pb isotope modelling generally considers many sources, and often the output from a model run could be insufficiently well-defined, sometimes with few clear major contributors. In the second theoretical example (Fig. 1e, Table 3) this is true, with most sources contributing less than 5%, and those with high contributions showing large ranges (e.g. Source 8, Table 3). At this point, we suggest further grouping *a posteriori*^65^. This combines raw contribution results in order to produce better-constrained relative contributions, but at the expense of specificity within sources. In practice, this means a user will not be able to discern between ore bodies but will be able to indicate more reliably which groups of ore bodies (or major mining regions) are major contributors. As with *a priori* grouping, this should be performed on groups which are isotopically similar or are located close to each other, allowing for meaningful conclusions to be drawn, despite the simplification this approach causes.

**Table 3:** Model outputs from theoretical example 2, displaying the raw MixSIAR output.

Raw Output	Mean (%)	2.5%	97.5%
Source 1	0.8	0.01	2.46
Source 2	0.8	0.01	2.81
Source 3	2.7	0.03	10.15
Source 4	1.8	0.02	5.31
Source 5	3.9	0.29	12.55
Source 6	2.9	0.14	11.42
Source 7	2.5	0.1	6.64
Source 8	48.5	4.59	63.78
Source 9	12.6	2.01	59.13
Source 10	1.5	0.11	5.54
Source 11	1.2	0.04	4.18
Source 12	1.1	0.01	3.87
Source 13	0.9	0.02	3.22
Source 14	17.8	2.15	28.74
Source 15	1.2	0.03	4.21

Mean values (in %) are displayed alongside the 95% credible intervals for each model.

To display the value of such an approach we have performed *a posteriori* analysis, combining sources which are isotopically similar, for minimising the number of possible end-members (see Table 3). For each possible mixture, all sources to be grouped should be summed for each individual solution^64^. In this way, a new distribution is created for the proportional contributions of the combined sources. The combination of sources 2, 11, 12, 13 and 15 in our theoretical example displays this (Table 4), with the contribution of this grouped source now observable, where before either the sources did not appear to contribute much to the mixture (e.g. Source 3 in Table 3) or displayed large possible ranges (e.g. Source 8 in Table 3). This approach has its advantages in clarifying broad source areas but it does remove some of the certainty to the approach requiring grouping which at times erases distinct sources. Nevertheless, without grouping, it may be difficult to say that any sources provided meaningful contributions to the Pb isotope signature, and so *a posteriori* grouping is a valuable tool when interpreting data which initially appears unconstrained.

**Table 4:** Model output from theoretical example 2, indicating output after grouping *a posteriori*.

*A posteriori* grouping	Sources Grouped	Mean (%)	2.5%	97.5%
Group 1	2, 11, 12, 13, 15	5.08	1.9	9.81
Group 2	3, 8, 9, 10	65.25	57.85	76.91
Group 3	4, 7	4.32	1.08	7.76
Group 4	5, 14	21.65	8.35	29.94
Group 5	1	0.8	0.01	2.46
Group 6	6	2.9	0.14	11.42

Mean values (in %) are displayed alongside the 95% credible intervals for each model.

This data, once produced, should be presented either as it is here, in table form (e.g. Table 3) and via figures (e.g. Fig. 1d), but always outlining the entire range of results^65^, and not simply the mean.

### Availability of materials and data

All lead isotope data used in this study were published previously and are summarized in tables in Supplementary Information. The model results generated during the current study are available from the corresponding author on request. The tool to allow for Bayesian modelling of a mixing envelope is an R code and can be downloaded from http://www.famer.unsw.edu.au/software/polygon.html. The MixSIAR model is an R package, which may be downloaded from https://cran.r-project.org/web/packages/MixSIAR/index.html.

## Results and Discussion

### Example 1: Pre-Anthropogenic Dust Sourcing

**Figure 2:**
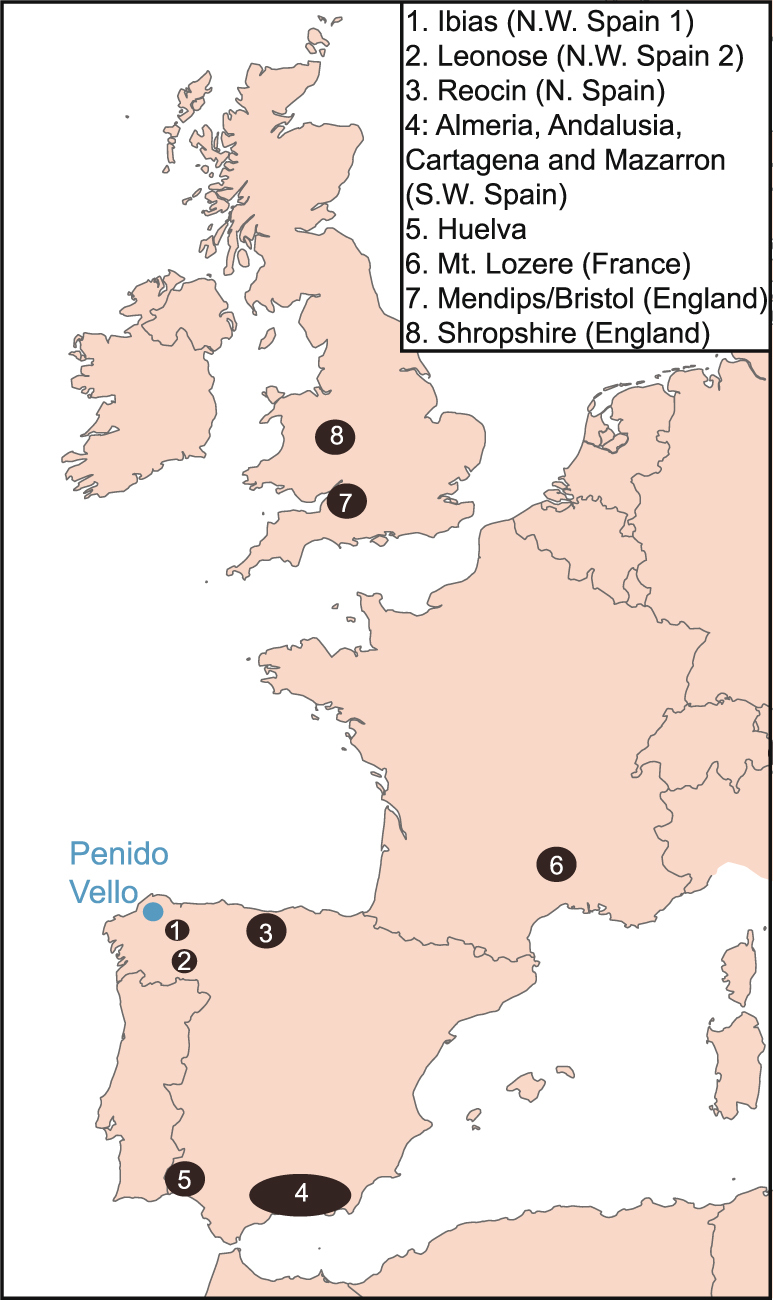
Map of western Europe displaying mining regions used to model contributions to Penido Vello bog (blue circle).

Due to the prevalence of anthropogenic Pb in the biogeochemical system since the inception of metallurgy, Pb isotopes are an imperfect tracer of natural fluxes during the recent past^67^. However, because of the ability of Pb isotopes to record source signatures, they have been applied to dust tracking studies^68^. Using a selection of natural geogenic sources, we attempt to indicate the source of pre-human Pb in the geochemical cycle within Penido Vello (PVO, Fig. 2), a peat bog in north-west Spain^52^, for the period 5000–1300 BCE.

**Figure 3:**
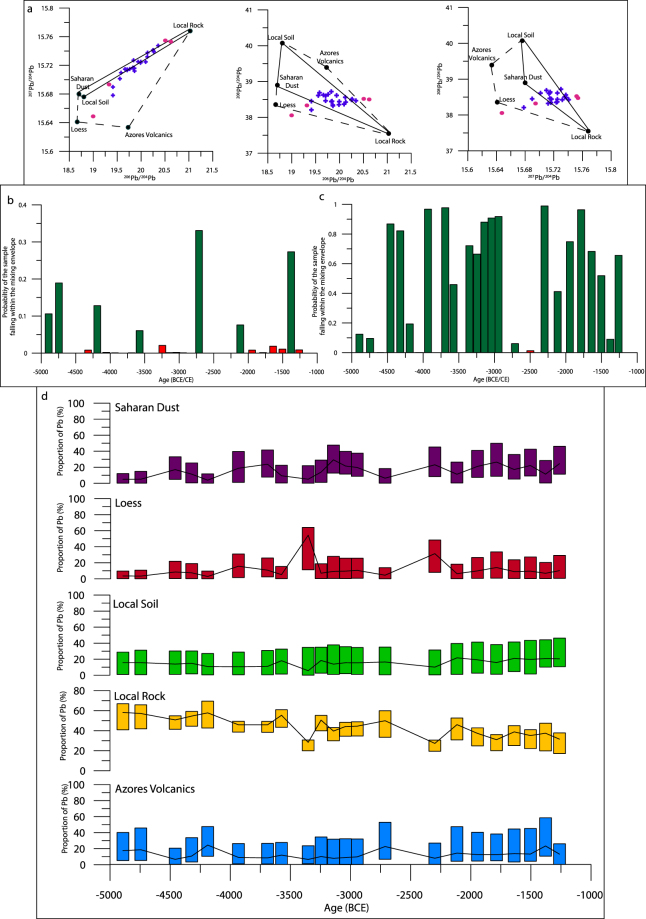
Convex hulls (**a**) from pre-anthropogenic dust tracing. Black circles denote sources used to reconstruct valid substrate mixtures from Penido Vello between 5000–1200 BCE^52^. Mixing envelopes as defined by the original publication are marked with solid black lines, whilst additional sources as suggested here result in mixing envelopes denoted by black dashed lines. Samples which fall within the convex hulls are marked in blue whilst those which fall outside one or more are pink, and have not been modelled. (**b**) Displays the output from the envelope mixing model using the original publications, (**c**) with additional sources. (**d**) Is the model output from the valid mixtures, displaying changing source contributions to the Pb contained within the mixture. In each case, the rectangle indicates the range of model outputs, with the upper and lower bounds signifying the 2.5% and 97.5% credible intervals, while black lines trace the mean value.

Using sources determined in the original publication (local rock, local soil^52^ and a Saharan dust value^69^, with the authors assuming negligible anthropogenic input), we have modelled the mixing envelope, indicating only a small number of samples fall inside (Fig. 3a,b). Moreover, a large number of samples (76%) fall a long way from the envelope, due to at least two unconsidered sources (Fig. 3a). As such, by estimating what the composition of these sources must be, and then comparing with published data from plausible sources, we determine two further inputs to the PVO site. The first of these, as speculated by the original publication, appears a volcanic input from the Azores, whilst the second is indicative of a loess-derived input. For demonstration purposes we use a value from a loess field in northern France to represent this source^70^, and data from a selection of lava and ash analysed in the Azores^71–73^. Modelling of the mixing space now indicates the majority of mixtures fall within (Fig. 3a,c), and so we have proceeded to model the relative inputs of the five sources. Since only five potential sources are considered, no grouping has been performed *a priori* (SI Table 1).

**Figure 4:**
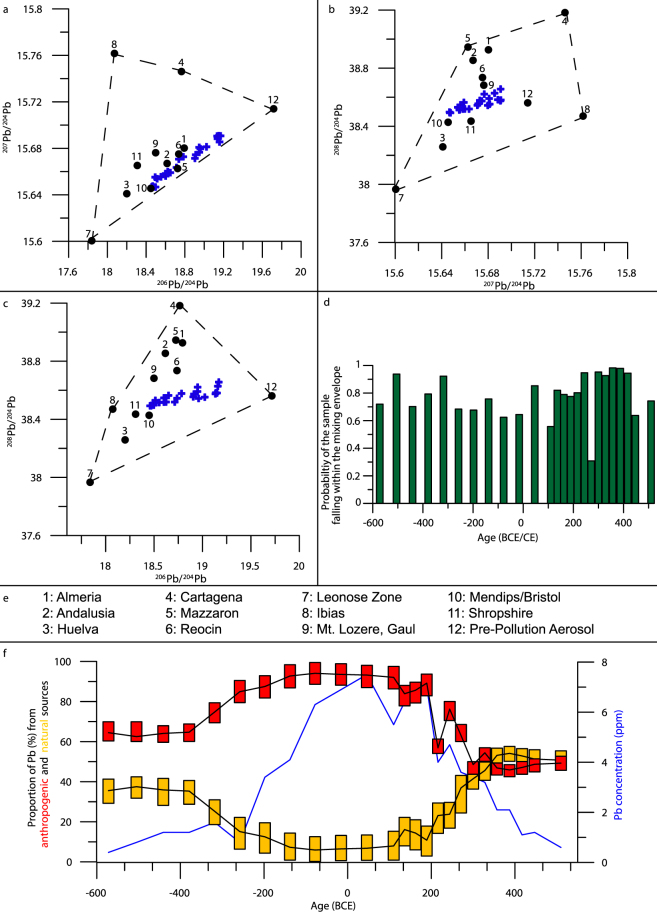
Convex hulls (**a**–**c**) from Penido Vello bog during the Roman period. As in a, black circles denote sources, with each labelled in panel 5e. Panel 5d displays the output from mixing envelope modelling, indicating all samples fall within the mixing space, and so there are no red crosses on panels 4a–c. 4 f is the model output from Penido Vello between 600 BCE and 550 CE. Red rectangles correspond to a posteriori grouping of all anthropogenic sources, while yellow rectangles are the modelled contribution for Pb from natural sources. Rectangles indicate upper and lower bounds as in Fig. 3, with black lines indicating the mean. Also shown (blue line) are the raw reported Pb concentrations for the core throughout this period^52^.

Using this new five-source model, a number of conclusions may be drawn. Samples appear to show periodic influx of Saharan dust into the site, with two main periods of higher (>15%) Saharan Pb proportions (3300–2800 BCE and 2100–1300 BCE) corresponding well with the original interpretation, where simple distance-based modelling indicated clear Saharan input midway (3300–2800 BCE)^52^. Outside of these time periods, the Pb isotope signal appears to be related to geogenic weathering, with local rock and soil the major Pb contributors throughout (Fig. 3d). A decreasing trend throughout the whole period for local rock (Fig. 3d) and increasing local soil proportions (Fig. 4c) appears indicative of local soil erosion related to farming and agriculture, with an increase in intensity throughout the time period, echoing evidence from pollen records^74^. The ability of our model to indicate a volcanic influence on the site is clear, with regular pulses in Azore-derived Pb (Fig. 3d).

### Example 2: Pollution Sourcing from Environmental Archives

The Roman period in Iberia was characterized by intense exploitation of natural resources. To disentangle the complex signals pertaining to possible anthropogenic sources of Pb to PVO during this period, we use a selection of potential sources (See SI Table 2), including a natural pre-pollution aerosol value, as determined by the authors^52^. Here, no *a priori* grouping was performed prior to modelling. Again, the location of the samples in the mixing envelope has been indicated graphically (Fig. 4a–c), and via a model (Fig. 4d), confirming all samples fall within the mixing envelope.

The first main point of interest is the changing contribution of natural (as represented by pre-pollution aerosol) and anthropogenic (the remainder) sources (Fig. 4f). Early in the period, the anthropogenic sources (all ore bodies) make up ~60% of the Pb, prior to increasing values over the period 350–200 BCE, before plateauing at roughly 90% between 100 BCE–150 CE (Fig. 4f). When compared to conclusions from the original publication, our data suggests a much higher proportion of Pb from anthropogenic sources, perhaps indicative of a previous underestimation of metal production. These values reflect the development and decline of the Roman metal industry, from the acquisition of Hispania during the Punic wars by 146 BCE^75^, through their exploitation, to the combined pressure of source exhaustion^2^ and repeated invasions of Hispania from 170 CE onwards^76^. The coherence of the modelled record with the raw Pb concentration data (Fig. 4f) indicates that much of the Pb deposited during this time period was anthropogenic, indicating values of ~90% for the anthropogenic fraction seem sensible.

**Figure 5:**
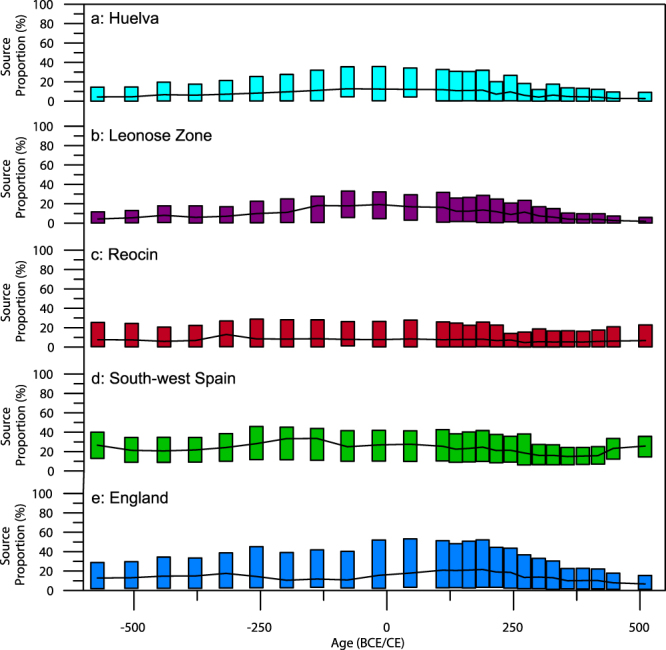
Model output from Penido Vello between 600 BCE and 550 CE, displaying the contribution of selected mining regions to the Pb mixture recorded in the sediments through this period. Again, rectangles indicate upper and lower bounds as in Fig. 3, with black lines indicating the mean.

Unfortunately, due to overlapping fields, many of the modelled ore-related proportion distributions are unconstrained. Some conclusions are still possible, with contributions from Almeria, Andalusia, Mazarron and Cartagena (after *a posteriori* grouping as south-west Spain), Huelva and the Leonese Zone peaking after the Punic wars (roughly 200 BCE), and falling away by 200 CE (Fig. 5), reflecting the developmental trend indicated by overall anthropogenic proportion changes (Fig. 5). Reocin (of the Basque-Cantabrian basin in Northern Spain) appears to peak just slightly before the Roman invasion (275 BCE), suggesting exploitation by the Iron Age Iberian cultures. Further exploitation of Reocin through the remainder of the period corroborates previous indications of mineral resource exploitation in the region during the Roman period^77,78^, evidence for which has been destroyed by more recent utilisation. Such conclusions indicate the value of such a modelling approach, allowing the user to interpret provenance in much finer detail, tracing pollution back to exact ore fields.

Interestingly, and previously unconsidered by the original publication, mining outside of Spain appears to have also had an impact on the isotopic mixture recorded here, with the contribution of the Mendip hills and Shropshire (grouped as England) appearing to change from very low contribution values at the start of the period, gradually rising after 10 CE, peaking by 150 CE and dropping off after 250 CE (Fig. 5). Such a profile reflects the well-constrained history of British lead mining, with evidence of mining in the Mendips from c.49 CE^79^, prior to considerable fluctuations in lead production throughout the period of Roman rule^80^.

### Example 3: Modelling of Artefact Provenance

**Figure 6:**
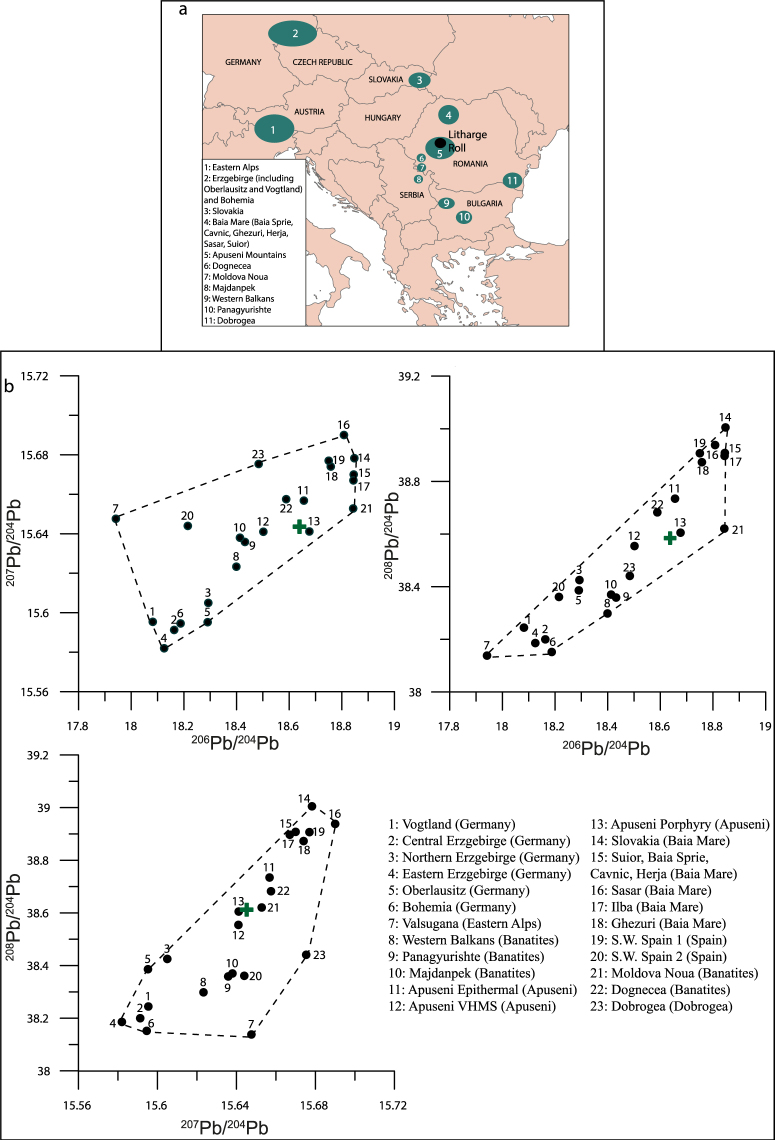
(**a**) Map of eastern Europe outlining the location of the Romanian artefact (Litharge roll, black circle). Also displayed are all mining regions modelled to understand the provenance of the Pb within the artefact. (**b**) displays convex hulls for modelling the litharge roll, Romania. As before, black circles reflect the sources used to reconstruct possible sources of Pb, whilst the green cross indicates the isotope composition of the modelled artefact.

Another potential application of MixSIAR and isotope mixing is the interpretation of the Pb isotope signal as recorded in individual metal artefacts. Determining the provenance of metal artefacts is of crucial importance in archaeology, and Pb isotopes have previously been used to this end in several studies^50,81,82^. Here we apply MixSIAR, in much the same way as done above, to the Pb isotope mixture of a litharge roll recovered from a Roman-age mining site in Romania^53^. To determine source contributions quantitatively, a dataset of potential sources has been assembled (SI Table 3, Fig. 6).

Due to the large number of sources, initial results indicate a wide range of potential origins for the Pb, but with Moldova Nouă ore field apparently the clearest single source (Table 5). To interpret the data more clearly, *a posteriori* grouping has been performed. Here, sources have generally been grouped by proximity (e.g. Baia Mare fields, and German fields), with others grouped by mineralisation and similar isotopic signature. An example of this is the inclusion of Apuseni Volcanogenic Massive Sulphides with other ores from the same mineralisation belt; the Banat Metallogenic Province^83^ such as Moldova Nouă and Majdanpek (Table 6, Fig. 6).

**Table 5:** Raw MixSIAR results for the litharge roll.

*A Priori* Group	Mean	2.50%	97.50%
Apuseni Epithermal	3.41%	0.10%	13.39%
Apuseni Porphyry	5.54%	0.14%	19.53%
Apuseni VMS	2.90%	0.08%	10.33%
Bohemia	2.12%	0.07%	7.48%
Central Erzgebirge	1.86%	0.05%	6.41%
Dobrogea	3.14%	0.08%	11.11%
Dognecea	3.03%	0.09%	10.49%
Eastern Erzgebirge	1.83%	0.05%	6.19%
Ghezuri	3.61%	0.09%	12.38%
Ilba	4.67%	0.15%	14.61%
Majdanpek	2.95%	0.07%	10.19%
Moldova Nouă	33.09%	21.73%	41.95%
Northern Erzgebirge	1.92%	0.04%	6.51%
Oberlausitz	1.96%	0.04%	7.19%
Pangyurishte	3.26%	0.08%	11.04%
SW Spain 1 (Almeria, Andalusia, Cartagena and Mazzarron)	2.91%	0.10%	10.71%
SW Spain 2 (Huelva)	2.22%	0.07%	7.60%
Săsar	3.64%	0.10%	12.12%
Slovakia	3.51%	0.08%	11.67%
Other Baia Mare	4.45%	0.14%	14.59%
Valsugana VMS (Eastern Alps)	1.37%	0.04%	4.64%
Vogtland	1.49%	0.03%	5.17%
Western Balkans	3.33%	0.10%	11.11%

Presented here are the source names, along with their modelled proportion of the Pb recorded in the artefact. Data are presented here as mean values, alongside lower and upper bounds, signifying the modelled 95% credible intervals.

**Table 6:** *A posteriori* grouping of raw MixSIAR results as displayed in Table 5, from the Romanian litharge roll.

*A Posteriori* Group	Included *a priori* groups	Mean	2.50%	97.50%
Apuseni	Apuseni Epithermal, Apuseni Porphyry	8.94%	1.14%	24.54%
Banatites	Apuseni VHMS, Dognecea, Moldova Nouă, Majdanpek, Panagyurishte, Western Balkans	48.56%	34.38%	60.98%
Germany	Bohemia, All Erzgerbirge (Central, Eastern. Northern), Oberlausitz, Vogtland	9.26%	3.55%	16.62%
Baia Mare	Ghezuri, Ilba, Săsar, Slovakia, Other Baia Mare	19.88%	9.77%	30.76%
Spain	SW Spain 1, SW Spain 2	6.92%	1.64%	16.19%
Eastern Alps	Valsugana VMS (Eastern Alps)	1.37%	0.04%	4.64%

After grouping sources as listed in Table 6, it can be stated that most Pb present in the artefact comes from the Banatites (average of nearly 50%) whilst Baia Mare Pb appears to constitute much of the remainder. Interestingly, the Apuseni group, which contains the Rosia Montana deposit where the litharge roll was discovered, does not contribute significantly (Table 6). This is very much in line with the conclusions reached in the original publication^53^, who suggested that the Pb present in the litharge roll was not from the Apuseni Mountains. Our model appears to suggest a Banat source as dominant, and within that, Moldova Nouă as the most likely mining field. Moldova Nouă, and its porphyry copper deposits^84^ has been exploited since Roman times, when Au and Ag were extracted from base metal ores^85^, with the typical method exploiting the chemical properties of litharge to bring the noble metals into the lead prior to further separation^53,86^.

It must be mentioned here that artefacts are much more likely to be composed of a one (or more) Pb ore source(s), in this case likely the Moldova Nouă ore field. The nature of the model, however is that it considers contributions from all sources to satisfy the mass balance, and therefore all sources may have a contribution, even when, as it appears here, the actual source is one ore field. As such, we suggest caution when interpreting such outputs. When one field dominates as it does here, we suggest that the user interprets this as a single, dominant source, and does not over-interpret other contributions. *A posteriori* grouping may be helpful at this point, as it is in this example, where the combination with other Banatitic ores leads to the clear domination of this signal (Table 6).

### Period pollution

One of the further advantages of MixSIAR is that it was developed to investigate populations of species, and not just individuals. As such it allows the user to analyse more than one mixture at a time and provide overall results for the dataset. In practice, this can allow a user not just to obtain proportions at discrete points in time, but to get an overall indication for an entire period. In the example used here, all mixtures have been run separately, and increases and decreases in certain sources may be observed. When all mixtures are run together, a much broader picture of pollution over a longer period may be produced. This is a faster process than modelling each individual mixture, and therefore may be useful for getting a better understanding of the dataset, or for comparing a few datasets covering a short period.

## Conclusions

Bayesian mixing models, as developed for food-web studies, may be applied to work focussing on understanding sources of pollution as documented by Pb isotope analyses. In a system where many sources are likely, they are a much more realistic approximation of the real world than the simple binary or ternary mixing currently utilised for such studies. Furthermore, they allow for incorporation of errors, and as such provide a more complex and exhaustive approach.

The model results, of course, depend on the quality of the data input. As such, it is vital thorough literature reviews are carried out prior to any analysis, and that all potential sources are considered. This is likely to produce very wide range values, for many sources. An *a posteriori* grouping approach as done here for the Roman period litharge roll may be applied to clarify what are likely to be the main sources, albeit at the cost of some specificity. Some systems, however, will not produce reliable or clear results. If the isotopic range for sources are similar or overlap significantly, the model may be unable to distinguish separate inputs to the mixture. In other cases, the existence of unknown and uncharacterised sources may result in the mixture falling outside the mixing envelope; in such cases, results are meaningless. A rigorous approach to source characterisation and model parametrisation should rule out errors such as these. Finally, we recommend using such models in tandem with more traditional provenance methods (e.g. 3-isotope plotting of all data); such a dual method approach is likely to produce the most reliable source characterisation. The few examples outlined here indicate the value of such an approach, first by clearly identifying which models require the consideration of further sources, or by identifying exact ore regions as major contributors to a mixture.

## Electronic supplementary material

Supplementary Information
