# Supplementary material for: Quantitative assessment of Pb sources in isotopic mixtures using a Bayesian mixing model
Source: Sci Rep. 2018 Apr 18;8:6154. doi: 10.1038/s41598-018-24474-0 (PMC5906678; doi:10.1038/s41598-018-24474-0)
Supplement: Supplementary file 1 — Supplementary Information [file 41598_2018_24474_MOESM1_ESM.doc]

# Quantitative assessment of Pb sources in isotopic mixtures using a Bayesian mixing model

Jack Longman^1,2*^, Daniel Veres^3^, Vasile Ersek^1^, Donald L. Phillips^4^, Catherine Chauvel^5,6^, Calin G. Tamas^7^

^1^ Department of Geography and Environmental Sciences, Northumbria University, Newcastle-upon-Tyne, NE1 8ST, United Kingdom

^2^ School of Ocean and Earth Sciences, University of Southampton, National Oceanography Centre, Waterfront Campus, Southampton, SO14 3ZH, United Kingdom

^3^ Romanian Academy, Institute of Speleology, Clinicilor 5, Cluj-Napoca, Romania

^4^ EcoIsoMix.com, Corvallis, Oregon, USA.

^5^ CNRS, Université Grenoble Alpes, Institut des Sciences de la Terre, UMR 5275 CNRS, Grenoble, France

^6^ Institut de Physique du Globe de Paris, Université Sorbonne Paris Cité, CNRS UMR 7154, Paris, France

^7^ Faculty of Biology and Geology, University Babeş-Bolyai, 1 M. Kogălniceanu str., 400084 Cluj-Napoca, Romania

*j.longman@soton.ac.uk

# Supplementary Information

*SI Table 1: Mean Pb isotopic compositions of potential sources from real-world example 1 (Pre-ant*hropogenic dust tracing, Penido Vello bog, Spain).

|  | **Example 1, Pre Anthropogenic Dust Tracing** | | | | | | | | |
| --- | --- | --- | --- | --- | --- | --- | --- | --- | --- |
|  | | **Average Isotope Composition** | | | **Standard Deviation** | | |  | **Reference** |
| **Source** | | ^206^Pb/^204^Pb | ^207^Pb/^204^Pb | ^208^Pb/^204^Pb | ^206^Pb/^204^Pb | ^207^Pb/^204^Pb | ^208^Pb/^204^Pb | n |
| **Local Soil** | | 18.803 | 15.676 | 40.073 | 0.0525 | 0.0185 | 0.4725 | 2 | 1 |
| **Local Rock** | | 21.028 | 15.768 | 37.549 | <0.0001 | <0.0001 | <0.0001 | 1 | 1 |
| **Saharan Dust** | | 18.7 | 15.68 | 38.9 | <0.0001 | <0.0001 | <0.0001 | 1 | 2 |
| **French Loess** | | 18.66 | 15.64 | 38.36 | <0.001 | <0.001 | <0.001 | 1 | 3 |
| **Azores Volcanics** | | 19.73 | 15.63 | 39.39 | <0.001 | <0.001 | <0.001 | 58 | 4–6 |

*SI Table 2: Mean* Pb isotopic compositions of potential sources from real-world example 2 (Roman period pollution in peat, Penido Vello bog, Spain).

|  | | **Example 2, Anthropogenic Pollution Tracing in Peat** | | | | | | | | |
| --- | --- | --- | --- | --- | --- | --- | --- | --- | --- | --- |
| **Source** | | | **Average Isotope Composition** | | | **Standard Deviation** | | |  | **Reference** |
| **Field** | ***A Priori* group (if applicable)** | | ^206^Pb/^204^Pb | ^207^Pb/^204^Pb | ^208^Pb/^204^Pb | ^206^Pb/^204^Pb | ^207^Pb/^204^Pb | ^208^Pb/^204^Pb | n |
| **Almeria** | S.W. Spain | | 18.793 | 15.680 | 38.927 | 0.050 | 0.019 | 0.063 | 23 | 7–9 |
| **Andalusia** | S.W. Spain | | 18.617 | 15.667 | 38.854 | 0.186 | 0.046 | 0.142 | 11 | 7,8 |
| **Cartagena** | S.W. Spain | | 18.765 | 15.746 | 39.182 | 0.009 | 0.014 | 0.011 | 2 | 7,8 |
| **Mazzarron** | S.W. Spain | | 18.725 | 15.663 | 38.945 | 0.006 | 0.009 | 0.027 | 7 | 7,8 |
| **Huelva** |  | | 18.201 | 15.641 | 38.258 | 0.021 | 0.009 | 0.045 | 10 | 7,10 |
| **Reocin** |  | | 18.737 | 15.675 | 38.736 | 0.017 | 0.010 | 0.028 | 11 | 11 |
| **Leonose Zone** |  | | 17.839 | 15.601 | 37.966 | 0.027 | 0.018 | 0.048 | 8 | 12 |
| **Ibias** |  | | 18.073 | 15.762 | 38.470 | 0.060 | 0.076 | 0.285 | 6 | 13 |
| **Gaul** |  | | 18.498 | 15.676 | 38.683 | 0.080 | 0.006 | 0.059 | 32 | 14 |
| **Mendips/**  **Bristol^a^** | England | | 18.309 | 15.665 | 38.435 | 0.135 | 0.024 | 0.133 | 50 | 15 |
| **Shropshire^b^** | England | | 18.449 | 15.646 | 38.428 | 0.035 | 0.006 | 0.001 | 12 | 15 |
| **Pre-Pollution Aerosol** |  | | 19.716 | 15.714 | 38.561 | 0.000 | 0.000 | 0.000 | 2 | 1 |

^a^ Isotope values from the database located in the counties Avon and Somerset

^b^ Isotope data from Shropshire Pb ores (not including Cu ores e.g. Malachite and Chalcopyrite)

*SI Table 3: Mean* Pb isotopic compositions of potential sources from real-world example 3 (Romanian artefact).

|  | **1C: Example 3, Artefact Tracing** | | | | | | | | | | | |
| --- | --- | --- | --- | --- | --- | --- | --- | --- | --- | --- | --- | --- |
| **Source** | | | **Average Isotope Composition** | | | | | **Standard Deviation** | | |  | **Reference** |
| ***A Priori Group*** | | **Fields Included** |  | ^206^Pb/^204^Pb | | ^207^Pb/^204^Pb | ^208^Pb/^204^Pb | ^206^Pb/^204^Pb | ^207^Pb/^204^Pb | ^208^Pb/^204^Pb | n |
| **Apuseni Epithermal** | | Baia de Arieș, Săcărâmb, Coranda, Roșia Montana | | | 18.6559 | 15.6568 | 38.7343 | 0.0142 | 0.0813 | 0.0030 | 41 | 16,17 |
| **Apuseni Porphyry** | | Roșia Poieni, Valea Morii | | | 18.6766 | 15.6411 | 38.6051 | 0.0087 | 0.0509 | 0.0022 | 7 | 16 |
| **Apuseni VMS** | | Vorța, Dealul Mare | | | 18.5020 | 15.6410 | 38.5545 | 0.0020 | 0.0185 | 0.0009 | 2 | 16 |
| **Bohemia** | | Horní Slavkov, Příbram-Vrancice, Kutná Hora | | | 18.1875 | 15.5945 | 38.1518 | 0.0200 | 0.0810 | 0.0021 | 11 | 18 |
| **Central Erzgebirge** | | Bärenstein, Annaberg, Měděnec, Marienberg, Hora Sv. Kateřiny | | | 18.1630 | 15.5913 | 38.2000 | 0.0248 | 0.1500 | 0.0070 | 12 | 18 |
| **Dobrogea** | | Altân Tepe, Somova | | | 18.4841 | 15.6754 | 38.4409 | 0.0998 | 0.0349 | 0.0946 | 5 | Unpublished data |
| **Dognecea** | | Dognecea | | | 18.5886 | 15.6575 | 38.6824 | 0.0020 | 0.0013 | 0.0040 | 2 | Unpublished data |
| **Eastern Erzgebirge** | | Freiberg, Sadisdorf, Zinnwald | | | 18.1250 | 15.5820 | 38.1857 | 0.0102 | 0.0903 | 0.0045 | 14 | 18 |
| **Ghezuri** | | Ghezuri | | | 18.7580 | 15.6740 | 38.8730 | 0.0064 | 0.0100 | 0.0342 | 4 | 16 |
| **Ilba** | | Ilba | | | 18.8440 | 15.6670 | 38.8970 | 0.0045 | 0.0058 | 0.0202 | 3 | 16 |
| **Majdanpek** | | Majdanpek | | | 18.4130 | 15.6380 | 38.3700 | <0.0001 | <0.0001 | <0.0001 | 1 | 19 |
| **Moldova Nouă** | | Moldova Nouă | | | 18.8428 | 15.6528 | 38.6206 | 0.0009 | 0.0006 | 0.0021 | 3 | Unpublished data |
| **Northern Erzgebirge** | | Schönborn, Gersdorf | | | 18.2928 | 15.6050 | 38.4250 | 0.0059 | 0.0675 | 0.0037 | 6 | 18 |
| **Oberlausitz** | | Ludwigsdorf | | | 18.2902 | 15.5952 | 38.3860 | 0.0031 | 0.0326 | 0.0019 | 5 | 18 |
| **Pangyurishte** | | Vlaikov Vruh, Elshitsa, Radka, Assarel, Medet, Chelopech, Elasite | | | 18.4319 | 15.6359 | 38.3586 | 0.0215 | 0.0793 | 0.0019 | 17 | 19,20 |
| **SW Spain 1** | | Almeria, Andalusia, Mazarron & Cartagena | | | 18.7500 | 15.6770 | 38.9070 | 0.1444 | 0.0367 | 0.1651 | 46 | 7,8 |
| **SW Spain 2** | | Huelva | | | 18.201 | 15.641 | 38.258 | 0.021 | 0.009 | 0.045 | 10 | 7,9 |
| **Sasar** | | Săsar | | | 18.8080 | 15.6900 | 38.9380 | 0.0143 | 0.0130 | 0.0471 | 3 | 16 |
| **Slovakia** | | Banska Stiavnica, Brehov, Rosalia | | | 18.8473 | 15.6782 | 39.0051 | 0.0020 | 0.0556 | 0.0033 | 5 | 21 |
| **Other Baia Mare** | | Baia Sprie, Cavnic, Herja, Suior | | | 18.8450 | 15.6700 | 38.9080 | 0.0236 | 0.0116 | 0.0381 | 20 | 16 |
| **Valsugana VMS (Eastern Alps)** | | Calceranica, Vetriolo, Valle Imperina | | | 17.9417 | 15.6476 | 38.1382 | 0.0144 | 0.0673 | 0.0024 | 24 | 22 |
| **Vogtland** | | Schönbrunn, Lauterbach, Gottesberg, Mühlleithen, Klingenthal | | | 18.0819 | 15.5954 | 38.2445 | 0.2511 | 0.0267 | 0.2611 | 11 | 18 |
| **Western Balkans** | | Sedmochlinesti, Chiprovitsi, Berkovitsa | | | 18.0819 | 15.5954 | 38.2445 | 0.2511 | 0.0267 | 0.2611 | 15 | 19,23,24 |

References

1. Kylander, M. E. *et al.* Refining the pre-industrial atmospheric Pb isotope evolution curve in Europe using an 8000 year old peat core from NW Spain. *Earth Planet. Sci. Lett.* **240,** 467–485 (2005).

2. Abouchami, W. & Zabel, M. Climate forcing of the Pb isotope record of terrigenous input into the Equatorial Atlantic. *Earth Planet. Sci. Lett.* **213,** 221–234 (2003).

3. Rousseau, D. D. *et al.* European glacial dust deposits: Geochemical constraints on atmospheric dust cycle modeling. *Geophys. Res. Lett.* **41,** 7666–7674 (2014).

4. Hildenbrand, A., Weis, D., Madureira, P. & Marques, F. O. Recent plate re-organization at the Azores Triple Junction: Evidence from combined geochemical and geochronological data on Faial, S. Jorge and Terceira volcanic islands. *Lithos* **210–211,** 27–39 (2014).

5. Elliott, T., Blichert-Toft, J., Heumann, A., Koetsier, G. & Forjaz, V. The origin of enriched mantle beneath São Miguel, Azores. *Geochim. Cosmochim. Acta* **71,** 219–240 (2007).

6. Beier, C., Haase, K. M., Abouchami, W., Krienitz, M.-S. & Hauff, F. Magma genesis by rifting of oceanic lithosphere above anomalous mantle: Terceira Rift, Azores. *Geochemistry, Geophys. Geosystems* **9,** n/a-n/a (2008).

7. Stos-Gale, Z., Gale, N., Houghton, J. & Speakman, R. Lead Isotope data from the Isotrace Laboratory, Oxford: Archaeometry Database 1, ores from the Western Mediterranean. *Archaeometry* **37,** 407–415 (1995).

8. Ruiz, C., Arribas, A. & Arribas, A. Mineralogy and geochemistry of the Masa Valverde blind massive sulphide deposit, Iberian Pyrite Belt (Spain). *Ore Geol. Rev.* **19,** 1–22 (2002).

9. Pomiès, C., Cocherie, A., Guerrot, C., Marcoux, E. & Lancelot, J. Assessment of the precision and accuracy of lead-isotope ratios measured by TIMS for geochemical applications: example of massive sulphide deposits (Rio Tinto, Spain). *Chem. Geol.* **144,** 137–149 (1998).

10. Velasco, F., Pesquera, A. & Herrero, J. M. Lead isotope study of Zn-Pb ore deposits associated with the Basque-Cantabrian basin and Paleozoic basement, Northern Spain. *Miner. Depos.* **31,** 84–92 (1996).

11. Velasco, F. *et al.* Geology and Geochemistry of the Reocín Zinc-Lead Deposit, Basque-Cantabrian Basin, Northern Spain. *Econ. Geol.* **98,** (2003).

12. Tornos, F., Ribera, F., Shepherd, T. J. & Spiro, B. The geological and metallogenic setting of stratabound carbonate-hosted Zn-Pb mineralizations in the West Asturian Leonese Zone, NW Spain. *Miner. Depos.* **31,** 27–40 (1996).

13. Arias, D. *et al.* Lead and sulfur isotope compositions of the ibias gold vein system (NW Spain): Genetic implications. *Econ. Geol.* **91,** 1292–1297 (1996).

14. Baron, S., Carignan, J., Laurent, S. & Ploquin, A. Medieval lead making on Mont-Lozere Massif (Cevennes-France): Tracing ore sources using Pb isotopes. *Appl. Geochemistry* **21,** 241–252 (2006).

15. Rohl, B. Lead isotope data from the isotrace laboratory, Oxford: Archaeometry data base 2, galena from Britain and Ireland. *Archaeometry* **38,** 165–180 (1996).

16. Marcoux, E., Grancea, L., Lupulescu, M. & Milési, J. Lead isotope signatures of epithermal and porphyry-type ore deposits from the Romanian Carpathian Mountains. *Miner. Depos.* **37,** 173–184 (2002).

17. Baron, S., Tǎmaş, C. G., Cauuet, B. & Munoz, M. Lead isotope analyses of gold-silver ores from Roşia Montanâ (Romania): A first step of a metal provenance study of Roman mining activity in Alburnus Maior (Roman Dacia). *J. Archaeol. Sci.* **38,** 1090–1100 (2011).

18. Niederschlag, E., Pernicka, E., Seifert, T. & Bartelheim, M. The Determination of Lead Isotope Ratios by Multiple Collector Icp-Ms: A Case Study of Early Bronze Age Artefacts and their Possible Relation With Ore Deposits of the Erzgebirge*. *Archaeometry* **45,** 61–100 (2003).

19. Amov, B. G. Lead isotope data for ore deposits from Bulgaria and the possibility for their use in archaeometry. *Berlin Beitr Archäom* **16,** 5–19 (1999).

20. Von Quadt, A. *et al.* The Elatsite porphyry copper deposit in the Panagyurishte ore district, Srednogorie zone, Bulgaria: U-Pb zircon geochronology and isotope-geochemical investigations of magmatism and ore genesis. *Geol. Soc. London, Spec. Publ.* **204,** 119–135 (2002).

21. Chernyshev, I. V., Chugaev, a. V. & Shatagin, K. N. High-precision Pb isotope analysis by multicollector-ICP-mass-spectrometry using 205Tl/203Tl normalization: Optimization and calibration of the method for the studies of Pb isotope variations. *Geochemistry Int.* **45,** 1065–1076 (2007).

22. Artioli, G., Angelini, I., Nimis, P. & Villa, I. M. A lead-isotope database of copper ores from the Southeastern Alps: A tool for the investigation of prehistoric copper metallurgy. *J. Archaeol. Sci.* **75,** 27–39 (2016).

23. Amov, B. G. Evolution of uranogenic and thorogenic lead, 1. A dynamic model of continuous isotopic evolution. *Earth Planet. Sci. Lett.* **65,** 61–74 (1983).

24. Stos-Gale, Z. A. *et al.* Lead isotope data from the Isotrace laboratory, Oxford: Archaeometry data base 5, ores from Bulgaria. *Archaeometry* **40,** 217–226 (1998).
